# Retraction: Emphysematous Cystitis: A Radiological Diagnosis of Potentially Life-Threatening Infection

**DOI:** 10.7759/cureus.r110

**Published:** 2024-01-25

**Authors:** Shouq A Alhusayni, Thekra H Alshammari, Abdullah A Althomali, Mishari M Alqahtani, Waad A Alanazi, Mohammed A Alghamdi, Faisal M AlDawsari, Elan M Homoud, Abdulrahman A Alharbi, Khalid E Althobaiti, Abdulrahman A Alsubaihi, Abdullah M Alsayed, Abdalmohsen M Albaqami, Ahmad M Alamer, Faisal Al-Hawaj

**Affiliations:** 1 College of Medicine, University of Hail, Hail, SAU; 2 College of Medicine, Taif University, Taif, SAU; 3 Family Medicine, Prince Sultan Military Medical City, Riyadh, SAU; 4 College of Medicine, Northern Border University, Arar, SAU; 5 College of Medicine, Medical University of Lodz, Lodz, POL; 6 College of Medicine, Majmaah University, Al Majma'ah, SAU; 7 College of Medicine, King Saud Bin Abdulaziz University for Health Sciences, Riyadh, SAU; 8 College of Medicine, King Abdulaziz University, Jeddah, SAU; 9 College of Medicine, Arabian Gulf University, Manama, BHR; 10 College of Medicine, Imam Abdulrahman Bin Faisal University, Dammam, SAU

The Editors-in-Chief have retracted this article. Concerns were raised regarding the identity of the authors on this article. Specifically, Faisal Alhaway and Malak Shammari have stated that they were added to this article without their knowledge or approval. The identity of the other authors could also not be verified. As the appropriate authorship of this work cannot be established, the Editors-in-Chief no longer have confidence in the results and conclusions of this article.

